# Construct a classification decision tree model to select the optimal equation for estimating glomerular filtration rate and estimate it more accurately

**DOI:** 10.1038/s41598-022-19185-6

**Published:** 2022-09-01

**Authors:** Zhenliang Fan, Qiaorui Yang, Zhuohan Xu, Ke Sun, Mengfan Yang, Riping Yin, Dongxue Zhao, Junfen Fan, Hongzhen Ma, Yiwei Shen, Hong Xia

**Affiliations:** 1 Nephrology Department, The First Affiliated Hospital of Zhejiang Chinese Medical University (Zhejiang Provincial Hospital of Traditional Chinese Medicine), 54 Youdian Road, Shangcheng District, Hangzhou, Zhejiang Province China; 2 Graduate School, Heilongjiang University of Chinese Medicine, No. 24 Heping Road, Xiangfang District, Harbin, Heilongjiang China; 3 Graduate School, Zhejiang Chinese Medical University, 548 Binwen Road, Binjiang District, Hangzhou, Zhejiang Province China; 4 Orthopedics Department, Shenzhen Hospital of Beijing University of Chinese Medicine (Longgang), 1 Dayun Road, Longgang District, Shenzhen City, Guangdong Province China

**Keywords:** Chronic kidney disease, Chronic kidney disease

## Abstract

Chronic kidney disease (CKD) has become a worldwide public health problem and accurate assessment of renal function in CKD patients is important for the treatment. Although the glomerular filtration rate (GFR) can accurately evaluate the renal function, the procedure of measurement is complicated. Therefore, endogenous markers are often chosen to estimate GFR indirectly. However, the accuracy of the equations for estimating GFR is not optimistic. To estimate GFR more precisely, we constructed a classification decision tree model to select the most befitting GFR estimation equation for CKD patients. By searching the HIS system of the First Affiliated Hospital of Zhejiang Chinese Medicine University for all CKD patients who visited the hospital from December 1, 2018 to December 1, 2021 and underwent Gate’s method of ^99m^Tc-DTPA renal dynamic imaging to detect GFR, we eventually collected 518 eligible subjects, who were randomly divided into a training set (70%, 362) and a test set (30%, 156). Then, we used the training set data to build a classification decision tree model that would choose the most accurate equation from the four equations of *BIS-2*, *CKD-EPI(CysC)*, *CKD-EPI(Cr-CysC)* and *Ruijin*, and the equation was selected by the model to estimate GFR. Next, we utilized the test set data to verify our tree model, and compared the GFR estimated by the tree model with other 13 equations. Root Mean Square Error (RMSE), Mean Absolute Error (MAE) and Bland–Altman plot were used to evaluate the accuracy of the estimates by different methods. A classification decision tree model, including BSA, BMI, 24-hour Urine protein quantity, diabetic nephropathy, age and RASi, was eventually retrieved. In the test set, the RMSE and MAE of GFR estimated by the classification decision tree model were 12.2 and 8.5 respectively, which were lower than other GFR estimation equations. According to Bland–Altman plot of patients in the test set, the eGFR was calculated based on this model and had the smallest degree of variation. We applied the classification decision tree model to select an appropriate GFR estimation equation for CKD patients, and the final GFR estimation was based on the model selection results, which provided us with greater accuracy in GFR estimation.

## Introduction

Chronic kidney disease (CKD) mainly refers to the irreversible damage of renal structure and (or) function caused by various etiological factors^1,2^. With the increasing prevalence of diabetes, hypertension, hyperuricemia and other diseases, the incidence of CKD is also rising with the passing years^3–7^. Some investigations have shown that nearly 120 million people suffer from CKD, which accounts for about 10.8% of the total population in China^8^, whereas the data in Europe and the United States is more severe^9^. However, when CKD progresses to End Stage Renal Disease (ESRD), the medical and social problems associated with it become more prominent, and CKD may become one of the leading causes of death ranking second to ischemic heart disease, stroke, infection and COPD by 2040^10,11^. Therefore, preventing the progression of CKD effectively has become a pressing medical problem.

Correct assessment of renal function in CKD patients is important to clinical treatment and the estimation of patients’ outcomes. As an indicator of glomerular filtration function, Glomerular Filtration Rate (GFR) is currently considered to be the most valuable parameter for assessing renal function in patients. However, measuring GFR in CKD patients is not a simple clinical procedure. At present the main method is to detect inulin, iohexol, ^125^I-iothalamate or Technetium-99m-diethylenetriaminepentaacetic acid (^99m^Tc-DTPA) and other exogenous markers dynamic clearance in the body to determine the GFR. Although inulin is considered as ‘the gold standard’ for determining GFR, this method is too complex and expensive to implement^12^. Gate’s method of ^99m^Tc-DTPA renal dynamic imaging, recommended by the Nephrology Committee of Society of Nuclear Medicine as well and served as a reliable method for determining GFR, was only used in some medical centers and could not cover all patients^13^. Therefore, various endogenous markers such as creatinine, cystatin C, and β2-microglobulin have been widely applied to assess renal function in patients, and various equations have been utilized to estimate Glomerular Filtration Rate (eGFR).

Although estimating eGFR via endogenous markers such as creatinine and cystatin C is a relatively conventional method, it is limited by the accuracy of the estimation equation. If eGFR deviates too much from the actual GFR, it will affect the clinician's judgment of the patient's condition and therapeutic regimen. For example, Yeli Wang et al. found that the CKD-EPI equation was not a reliable estimate of GFR in the South Asian population^14^. Marco van Londen et al. in a study of living kidney donors found that none of the current eGFR estimation equation can accurately estimate the donor's GFR, which is likely to underestimate the true rate of decline in GFR in 3 months–5 years after donation^15^. In addition, some equations customized for specific ethnic groups also showed huge deviations in the validation population in subsequent studies^16^.

Therefore, we believe that developing new equations for specific crowds or based on new endogenous markers may not be an optimal solution. In this study, we attempted to construct a classification decision tree model to select a more appropriate eGFR equations for CKD patients, and obtaining a more accurate estimate of glomerular filtration rate.

## Results

### Clinical characteristics and demographic data of the patients

We eventually collected 518 eligible subjects, 70% (362) of whom were assigned to training set to construct the discriminant model and 30% (156) to test set to verify whether the model is accurate or reliable. In this study, most CKD patients were over 50 years old, with an average age of 60.63. The number of males was slightly larger than females, accounting for 61.88% in the training set and 66.67% in the test set. However, kidney transplant patients were excluded in this study. Only about one-third of patients had a renal biopsy with a definite renal pathology diagnosis, and half of them were diagnosed with diabetic nephropathy. Among primary glomerular diseases, IgA nephropathy accounts for the highest proportion, which is about 20% of all pathologically confirmed patients. Our study also included a small subset (8.11%) of CKD patients, who were taking calcium dobesilate. They were always excluded from the development of GFR estimation equations because calcium dobesilate interferes with creatinine measurement and causes overestimation of glomerular filtration rate. Details of patients’ clinical and demographic data are shown in Table 1.

**Table 1:** Clinical and demographic data of patients (Mean (P_25_–P_75_)).

	Training set (n = 362)	Test set (n = 156)	Total population (n = 518)
Age (year)	60.2 (50–71)	61.7 (52–72)	60.6 (51–71)
Male	224 (61.9%)	104 (66.7%)	328 (63.3%)
Height (cm)	167.6 (158–170)	164.2 (158–170)	166.6 (158–170)
Weight (kg)	68.8 (55.6–72.2)	65.4 (57.0–77.0)	67.8 (55.9–73.0)
Body surface area (m^2^)	1.7 (1.6–1.8)	1.7 (1.6–1.9)	1.7 (1.6–1.8)
BMI	25.4 (21.3–26.6)	24.2 (21.8–26.3)	25.0 (21.5–26.5)
SBP (mmHg)	142.2 (126.0–157.0)	147.3 (128.3–164.0)	143.7 (127.0–158.3)
DBP (mmHg)	80.2 (70.0–88.3)	79.2 (68.0–89.0)	79.9 (69.0–89.0)
Smoking	68 (18.8%)	32 (20.5%)	100 (19.3%)
Drinking	46 (12.7%)	26 (16.7%)	72 (13.9%)
Unilateral nephrectomy	6 (1.7%)	8 (5.1%)	14 (2.7%)
**Hypertension**			
Grade 1	75 (20.7%)	24 (15.4%)	99 (19.1%)
Grade 2	69 (19.1%)	36 (23.1%)	105 (20.3%)
Grade 3	160 (44.2%)	82 (52.6%)	242 (46.7%)
Diabetes	104 (28.7%)	60 (38.5%)	164 (31.7%)
Cardiovascular disease	43 (11.9%)	28 (18.0%)	71 (13.7%)
Cerebral hemorrhage	3 (0.8%)	2 (1.3%)	5 (1.0%)
Cerebral infarction	28 (7.7%)	9 (5.8%)	37 (7.1%)
Hyperuricemia	67 (18.5%)	28 (18.0%)	95 (18.3%)
Gout	45 (12.4%)	21 (13.5%)	66 (12.7%)
Edema	130 (35.9%)	68 (43.6%)	198 (38.2%)
**Renal pathology**			
MCD	1 (0.3%)	0 (0%)	1 (0.2%)
IgAN	30 (8.3%)	7 (4.5%)	37 (7.1%)
MsPGN	13 (3.6%)	1 (0.6%)	14 (2.7%)
MPGN	1 (0.3%)	0 (0%)	1 (0.2%)
MN	5 (1.4%)	1 (0.6%)	6 (1.2%)
FSGS	2 (0.6%)	1 (0.6%)	3 (0.6%)
Hypertensive kidney lnjury	10 (2.8%)	6 (3.9%)	16 (3.1%)
Diabetic nephropathy	44 (12.2%)	30 (19.2%)	74 (14.3%)
HSP	2 (0.6%)	0 (0%)	2 (0.4%)
Lupus nephritis	3 (0.8%)	2 (1.3%)	5 (1.0%)
Hyperuricemic nephropathy	13 (3.6%)	6 (3.9%)	19 (3.7%)
Polycystic kidney	4 (1.1%)	0 (0%)	4 (0.8%)
Without renal biopsy	234 (64.6%)	102 (65.4%)	336 (64.9%)
Glucocorticoid	30 (8.3%)	14 (9.0%)	44 (8.5%)
Immunosuppressor	47 (13.0%)	14 (9.0%)	61 (11.8%)
Diuretic	136 (37.6%)	66 (42.3%)	202 (39.0%)
Uric acid lowering therapy	212 (58.6%)	83 (53.2%)	295 (57.0%)
SGLT2i	3 (0.8%)	6 (3.9%)	9 (1.7%)
RASi	117 (32.3%)	45 (28.9%)	162 (31.3%)
Statin	132 (36.5%)	58 (37.2%)	190 (36.7%)
Calcium dobesilate	32 (8.8%)	10 (6.4%)	42 (8.1%)
White blood cell (× 10^9^)	6.0 (4.5–7.0)	6.28 (4.9–7.2)	6.1 (4.7–7.0)
Red blood cell (× 10^9^)	4.3 (2.9–3.9)	5.4 (2.8–3.8)	4.6 (2.9–3.9)
Hemoglobin (g/L)	102.3 (87.0–118.0)	98.6 (82.0–113.8)	101.2 (86.0–116.0)
Platelet (× 10^9^)	185.4 (141.0–221.0)	188.1 (140.5–225.5)	186.2 (141.0–222.3)
C-Reactive protein (mg/L)	9.0 (1.0–4.9)	7.7 (1.0–5.0)	8.6 (1.0–4.9)
AG (mmol/L)	8.4 (4.1–12.4)	9.4 (6.1–13.0)	8.7 (4.6–12.6)
Lactic acid (mmol/L)	1.3 (0.9–1.5)	1.3 (0.9–1.5)	1.3 (0.9–1.5)
Osmotic pressure (mosm/L)	283.2 (280.0 -289.0)	283 (280.6–290.4)	283.2 (280.1–289.6)
FPG (mmol/L)	5.1 (4.2–5.3)	5.2 (4.3–5.6)	5.2 (4.2–5.4)
Glycated hemoglobin (%)	6.0 (5.3–6.3)	6.0 (5.3–6.5)	6.0 (5.3–6.3)
Serum potassium (mmol/L)	7.4 (3.9–4.6)	4.3 (3.9–4.6)	6.5 (3.9–4.6)
Serum sodium (mmol/L)	141.0 (139.4–142.5)	140.4 (139.9–143.0)	140.8 (139.5–142.7)
Serum calcium (mmol/L)	3.1 (2.1–2.3)	2.2 (2.1–2.3)	2.8 (2.1–2.3)
Serum phosphate (mmol/L)	2.4 (1.1–1.6)	1.5 (1.2–1.6)	2.1 (1.1–1.6)
Triglyceride (mmol/L)	1.8 (1.1–2.1)	3.6 (1.1–2.2)	2.4 (1.1–2.1)
Total cholesterol (mmol/L)	4.5 (3.6–5.2)	4.4 (3.5–5.1)	4.5 (3.5–5.2)
High-density lipoprotein (mmol/L)	1.1 (0.9–1.3)	1.1 (0.9–1.3)	1.1 (0.9–1.3)
Llow-density lipoprotein (mmol/L)	2.3 (1.7–2.9)	2.3 (1.6–2.8)	2.3 (1.7–2.8)
Total serum protein (g/L)	62.5 (57.4–67.4)	62.3 (57.8–67.1)	62.4 (57.6–67.3)
Aalbumin (g/L)	35.1 (32.0–38.9)	35.0 (31.9–39.1)	35.1 (32.0–38.9)
Globulin (g/L)	27.4 (24.0–30.5)	27.2 (23.7–29.6)	27.3 (24.0–30.1)
Prealbumin (mg/L)	287.4 (241.0–336.0)	285.3 (235.5–342.0)	286.8 (237.8–336.3)
Homocysteine (μmol/L)	26.7 (15.9–28.3)	27.0 (16.3–29.7)	26.8 (16.0–29.1)
**PRO**			
−	76 (21.0%)	28 (18.0%)	104 (20.1%)
±	33 (9.1%)	20 (12.8%)	53 (10.2%)
+	80 (22.1%)	32 (20.5%)	112 (21.6%)
++	122 (33.7%)	48 (30.8%)	170 (32.8%)
+++	51 (14.1%)	28 (18.0%)	79 (15.3%)
Urine α1 microglobulin (mg/L)	42.4 (16.3–63.3)	59.6 (22.3–70.8)	47.6 (18.0–64.1)
Urine β2 microglobulin (μg/L)	8891.3 (183.6–10,928.0)	6720.6 (111.7–7788.1)	8237.6 (172.9–10,512.7)
Microalbuminuria (mg/L)	875.2 (74.6–1248.6)	962.9 (61.0 -1378.3)	901.6 (73.1–1303.8)
Urine NAG-enzyme (u/L)	12.4 (6.5–14.2)	11.6 (6.7–14.3)	12.1 (6.6–14.2)
Urinary albumin creatinine ratio (mg/umol)	0.2 (0.01–0.23)	0.2 (0.01–0.29)	0.2 (0.01–0.25)
24-hour urine volume (L)	6.3 (1.2–2.1)	1.7 (1.2–2.1)	4.9 (1.2–2.1)
24-hour urinary protein quantity (mg)	1818.9 (221.0–2665.0)	2020.4 (231.0–2975.0)	1879.0 (224.0–2781.9)
B-type natriuretic peptide (ng/L)	195.0 (19.0–132.1)	193.7 (27.9–179.4)	194.6 (20.4–148.2)
Free triiodothyronine (pmol/L)	4.5 (3.0–4.0)	3.3 (2.9–3.8)	4.1 (3.0 -3.9)
Free thyroxine (pmol/L)	12.6 (11.6–14.1)	12.1 (11.0–13.9)	12.4 (11.4–14.1)
Thyroid stimulating hormone (mIU/L)	5.3 (1.1–2.7)	3.7 (1.0 -2.5)	4.8 (1.0–2.7)
Parathyroid hormone (μg/mL)	89.1 (11.4–136.1)	99.9 (13.5–142.2)	92.3 (12.1–137.3)
Ferroprotein (ng/mL)	226.2 (85.1–279.0)	255.0 (98.7–323.2)	235.0 (89.6–292.8)
Uric acid (umol/L)	437.9 (347.8–518.3)	453.2 (363.8–532.5)	442.5 (349.0–525.0)
Serum creatinine (umol/L)	312.5 (146.5–424.8)	371.2 (174.3–493.5)	330.2 (150.8–451.0)
Urea nitrogen (mmol/L)	17.1 (9.0–21.5)	18.1 (9.5–22.8)	17.4 (9.1–21.9)
Cystatin C (mg/L)	5.6 (2.0–3.6)	4.1 (2.0–3.7)	5.6 (2.0–3.7)
Glomerular filtration rate (mL/min)	28.3 (15.3–38.0)	26.3 (14.8–34.0)	27.7 (15.2–36.8)

### Classification decision tree model and variable importance

**Figure 1:**
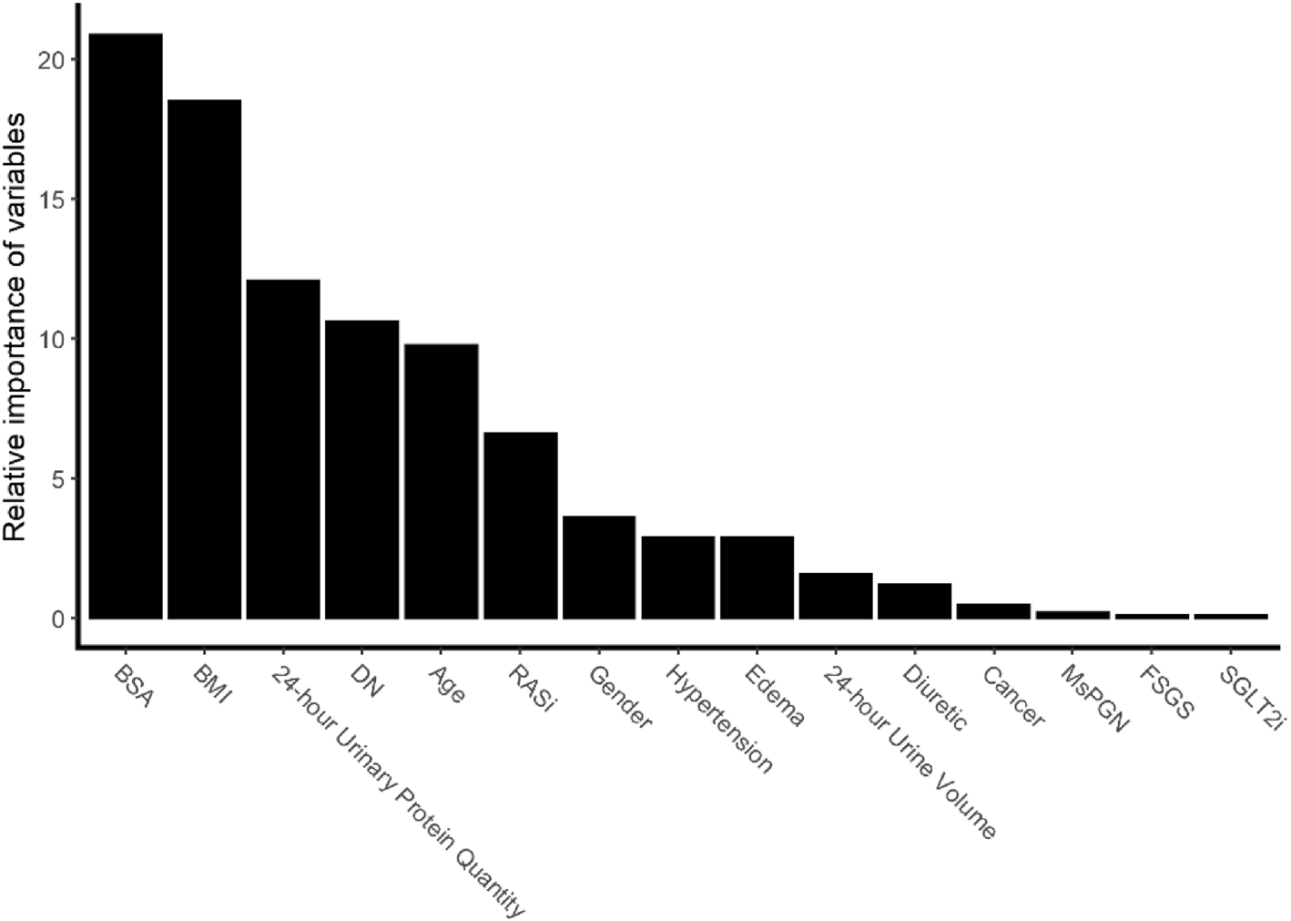
Relative importance of variables.

A total of 28 variables, including patient demographic data, past medical history, medication status, renal pathology results and laboratory measurement, were used to construct a classification decision tree model. Then we filtrated 15 relatively important variables and further selected the relative key variables to build the final classification decision tree model (Fig. 1).

**Figure 2:**
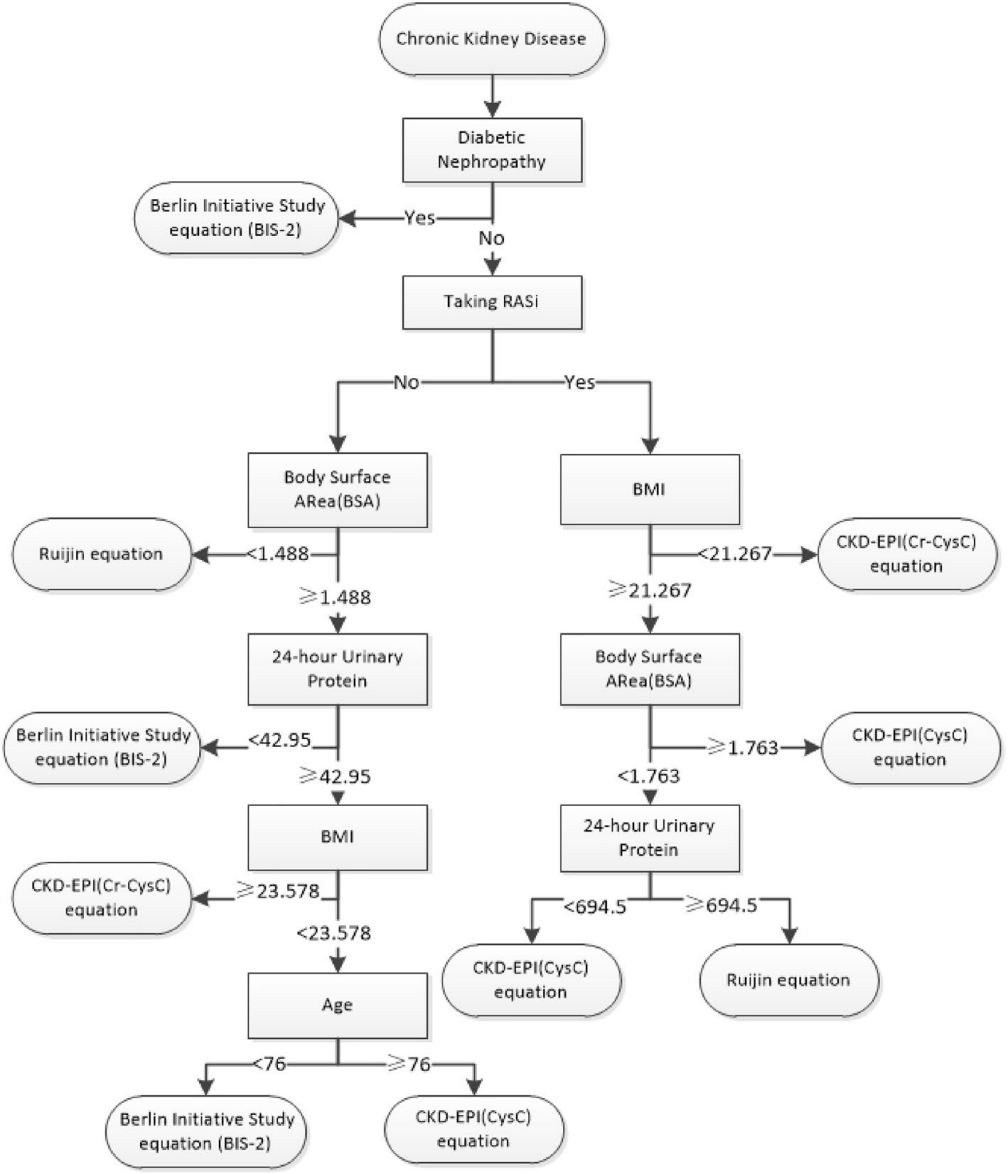
Classification decision tree model.

BSA, BMI, 24-hour urine protein quantity, diabetic nephropathy, age and RASi were selected to constitute the final classification decision tree model. As shown in Fig. 2, when a patient with CKD was diagnosed with diabetic nephropathy, *BIS-2* equation was recommended to him directly, otherwise he would need to be assessed based on RASi, BSA, 24-hour urine protein quantitation, and age. RASi may be a key factor influencing GFR estimation in patients with non-diabetic nephropathy. Meanwhile, patients with low BSA or BMI may be more appropriate to use *Ruijin* equation or *CKD-EPI (Cr-CysC)* equation. *CKD-EPI (CysC)* equation may be more suitable for CKD patients with higher BMI and BSA, which may be related to the fact that cystatin C is less affected by metabolic factors than creatinine.

### Classification decision tree model estimation of GFR performance

We estimated GFR by 13 known equations and the classification decision tree model respectively. Then, the results obtained above were compared with the GFR converted by 1.73 m^2^ standard body surface area. Finally, we found that the estimated GFR of *Ruijing*, *BIS-2*, *CKD-EPI*, *abbreviated MDRD* and classification decision tree model approximated to the levels measured by ^99m^Tc-DTPA (Table 2, GFR estimated by tree model and traditional equations for the training set and total population are shown in Supplement 1).

**Table 2:** GFR estimated by decision tree model and traditional equations based on BSA for test set (Mean (P_25_–P_75_)).

Equation^a^	Test set (n = 156)
Cockcroft gault	64.6 (30.7–85.9)
MDRD	59.9 (25.42–84.78)
Abbreviated MDRD	23.4 (9.9–33.69)
Chinese modification MDRD	72.0 (30.6–101.9)
Chinese modification abbreviated MDRD	30.6 (12.9–43.9)
CKD-EPI(Cr)	23.9 (9.3–33.4)
CKD-EPI(CysC)	24.2 (13.2–28.8)
CKD-EPI(Cr-CysC)	22.7 (10.7–29.9)
Asian modified CKD-EPI(Cr)	25.2 (9.8–35.3)
BIS-2	27.6 (15.8–34.2)
MacIsaac	31.3 (19.3–38.7)
Ruijin	28.2 (14.6–38.2)
Xiangya	38.8 (27.1–49.0)
Decision tree classifier	25.3 (13.6–32.1)
sGFR^b^	26.6 (15.1–34.6)

^a^All GFR estimation equations were converted to a uniform unit, mL/min per 1.73 m^2^.

^b^sGFR: GFR was measured by ^99m^Tc-DTPA, and the GFR was converted to 1.73 m^2^ standard body surface area based on the patient's body surface area.

RMSE and MAE were used to evaluate the accuracy of these equations and the classification decision tree model in estimating GFR. According to RMSE and MAE, the values of *Ruijin*, *BIS-2* and *CKD-EPI (Cr-CysC)* were far less than the other 10 equations, indicating a more accurate estimation (Table 3). However, our classification decision tree model combined the accurate prediction of *BIS-2*, *CKD-PEI (CysC)*, *CKD-EPI (Cr-CysC)* and *Ruijin* equations for specific population, showing a more precise estimation of GFR for CKD patients. MAE can better represent the accuracy of estimating GFR by the model, and the MAE of the classification decision tree model in the test set was only 8.5, which was much lower than other estimation equations (RMSE and MAE for the training set and total population are shown in Supplement 1).

**Table 3:** RMSE and MAE of various estimation equations.

	RMSE	MAE
	Test set (n = 156)	Test set (n = 156)
Cockcroft gault	50.4	38.7
MDRD	48.8	35.0
Abbreviated MDRD	12.3	9.6
Chinese modification MDRD	63.7	46.3
Chinese modification abbreviated MDRD	15.9	11.7
CKD-EPI(Cr)	12.7	9.9
CKD-EPI(CysC)	14.0	9.5
CKD-EPI(Cr-CysC)	12.8	9.3
Asian modified CKD-EPI(Cr)	13.2	10.1
BIS-2	11.9	8.8
MacIsaac	15.0	10.8
Ruijin	11.6	8.9
Xiangya	16.2	13.9
Decision tree classifier	12.2	8.5

### Comparison of deviation of various methods for estimating GFR

**Figure 3:**
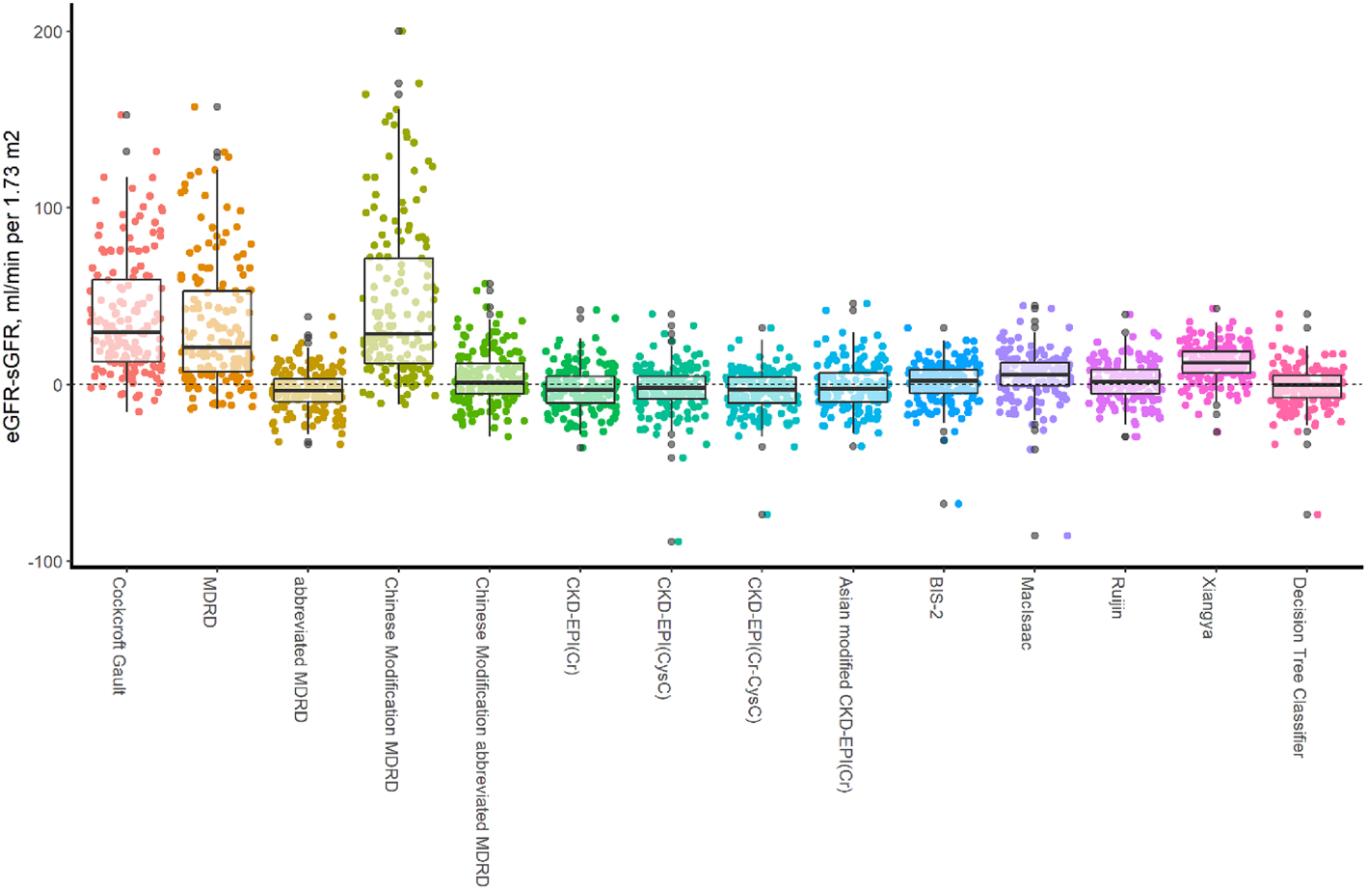
Variations in estimates of GFR in different equations of test data. (**a**) eGFR: Glomerular filtration rate was estimated based on an equation or model; (**b**) sGFR: GFR was measured by ^99m^Tc-DTPA, and the GFR was converted to 1.73 m^2^ standard body surface area based on the patient's body surface area.

In the box plot (Fig. 3), the estimation accuracy of *Cockcroft Gault*, *MDRD* and *Chinese Modification MDRD* was far worse than that of others, which tended to overestimate the glomerular filtration rate of CKD patients. However, over-optimistic estimates of renal function in CKD patients might seriously affect clinical decision making and bring unpredictable risks to patients. Although the average deviation of other equations was not remarkably different from the classification decision tree model, the estimation bias obtained from classified decision trees was more centralized (Variations in eGFR in different equations of training data and total population are shown in Supplement 1).

### Comparison of degree of variation in GFR estimation bias

**Figure 4:**
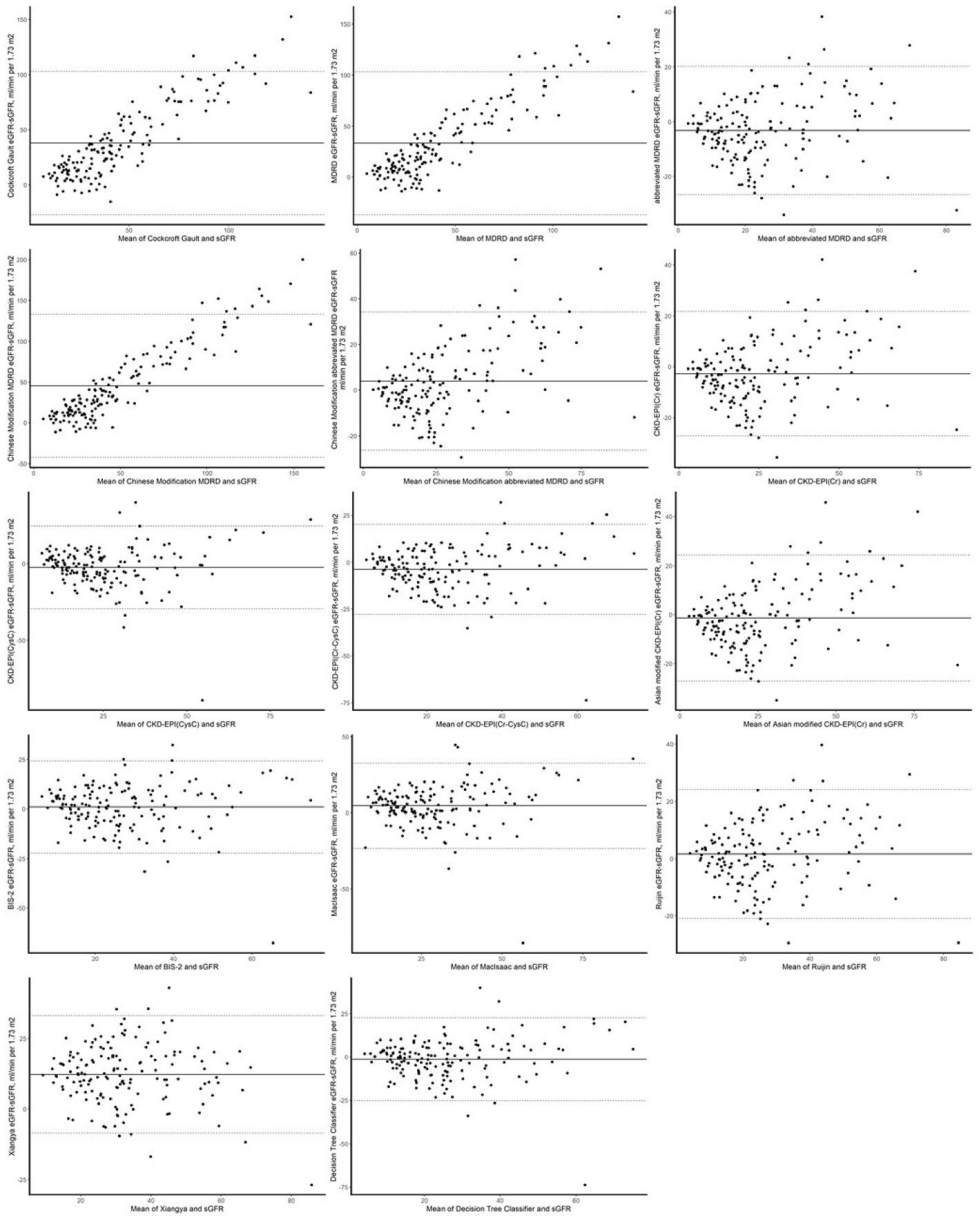
Bland–Altman diagram for the test set. (**a**) eGFR: Glomerular filtration rate was estimated based on an equation or model; (**b**) sGFR: GFR was measured by ^99m^Tc-DTPA, and the GFR was converted to 1.73 m^2^ standard body surface area based on the patient's body surface area.

According to Bland–Altman plot, the eGFR based on the classification decision tree model maintained a high degree of consistency among different patients. Bland–Altman diagram also showed that GFR estimation of small RMSE and MAE, such as *BIS-2*, *Ruijin* and *abbreviated MDRD*, had a large deviation (Fig. 4, Bland–Altman plots for the training set and total population are shown in Supplement 1).

## Discussion

Chronic kidney disease has become a worldwide public health problem, and most patients with CKD experience the irreversible renal impairment and end up with ESRD^1^. Protecting the renal function of patients is one of the prerequisites to improve the prognosis of CKD patients. In addition, the kidney is also a major organ for drug metabolism and excretion, which means that the variety and dosage of drugs will be adjusted according to renal function in CKD patients. Therefore, accurate assessment of renal function in CKD patients is critical.

As the most reliable indicator of renal function, GFR is of great significance for patients and is also the best choice for clinicians to make clinical decision^17^. But the actual measurement of GFR is a very complex operation. Most of the time, clinicians only estimate GFR by combining serum levels of endogenous markers such as creatinine and cystatin C with GFR estimation equations. However, this will raise a wholly new problem—estimation bias, which may be unacceptable and lead to misjudgments of patient outcomes^18^. How to reduce eGFR bias has always been a topic of interest to nephrologists. Previous studies mainly focused on developing new estimation equations or finding new endogenous markers^16,19^. In this study, we tried to integrate several equations, and used the classification decision tree model to determine the optimal equation for different patients, and finally obtained a more accurate eGFR.

There are many explanations for this estimation bias, which can be divided into two categories^20^. First, some eGFR equations are seriously over-fitting. They show high accuracy in the populations who have been developed already, but lose robustness in the population beyond the base value range. Currently, more than 80% of GFR estimation equations are based on Caucasian or black clinical data, while only a small percentage of equations include data from Asian populations, which may have a large bias in the population of Asian countries such as China and Japan or other ethnic minority areas^14,21–24^. In addition to race and genetic specificity, the variation of patients' disease status is also one of the main sources of equations bias. Hyperperfusion and hyperfiltration of the glomerulus are common in obese or diabetic patients, which may also lead to inaccurate estimates^25,26^. Bassiony et al.^27^ found that all commonly used formulae for GFR estimation were not accurate enough in morbidly obese patients. And only the 24-hour creatinine excretion rate can be used to estimate renal function indirectly. The unique pathophysiological and hemodynamic characteristics of patients with obstructive nephropathy or transplantation also make many GFR estimation equations unsuitable for them^15,28^.

Second, many equations rely on a single endogenous marker, whose serum concentration is likely to be affected by other factors, to estimate GFR. For example, calcium hydroxybenzene sulfonate is applied to many CKD patients with diabetic peripheral vascular disease, which can markedly affect serum creatinine measurement and lead to a significant overestimation of eGFR in all creatinine-based eGFR estimation equations. In addition, factors such as patient muscle mass, diet, exercise and metabolic level can interfere with endogenous marker levels, attributing to a bias in the estimation of GFR. Pottel et al. developed an equation based on creatinine, which was more accurate than others. However, this equation has a large bias in patients with reduced creatinine production, such as anorexia, paralysis, malnutrition, proteinuria, and hypoalbuminemia^29^. What’s more, Xie et al. also proposed that inflammatory state and thyroid function could also affect the estimation of GFR^26^.

Therefore, researchers attempted to use novel endogenous filtration markers, combined with multiple endogenous markers, to develop eGFR estimation equations for specific populations or a multi-parameter estimation equation^30–32^. Unfortunately, these methods do not provide more accurate estimates for GFR. Li et al. developed a *Xiangya* GFR estimation equation for Chinese CKD population and showed much more efficacy than EPI equation in the validation data^19^. However, this equation did not show strong robustness in subsequent studies^16^. The *MDRD* is a classical multi-parameter equation that includes the creatinine, urea nitrogen, age and serum albumin levels of CKD patients. However, Hu^33^ and our results showed that the accuracy of *MDRD* or *Chinese modified MDRD* is not ideal. What’s more, adding too many parameters into the equation increases the complexity of the equation, which is not convenient for clinicians^33^.

We believe that overfitting and interference from non-renal factors may be present in any GFR estimation equations, and developing a new equation may not eliminate these two problems fundamentally^17,18^. New equation or correction coefficients have been developed for many countries and ethnic population. For example, *Ruijin* and *Xiangya* equations developed for Chinese are greatly improved on *CKD-EPI* and *MDRD* equations developed for Caucasian and black people. Instead of putting the same equation into all CKD patients, we try to combine multiple equations and choose a suitable and accurate GFR estimate equation for each CKD patient. Therefore, we attempted to utilize machine learning to predict the most accurate GFR estimation equation for each CKD patient and then to estimate GFR. We chose the classification decision tree model to implement this process. Our classification decision tree model indicates that BSA, BMI, 24-hour urine protein quantity, diabetic nephropathy, age and RASi may be vital factors for GFR estimation bias. To achieve a more accurate estimate of GFR, the classification decision tree model was used to classify CKD patients according to the variables above and then to select the optimal estimation equation for them to minimize the bias. What we eventually obtained from this research was that the values of MAE and RMSE based on classified decision tree model were far smaller than other 13 equations, which verified our hypothesis. This indicated that the classification decision tree model could combine the advantages of multiple equations and automatically select the appropriate equation to obtain more accurate eGFR.

However, there are some limitations in the study. First, the sample size of this study is relatively limited. The ^99m^Tc-DTPA measurement of GFR in CKD patients is not a clinical procedure that can be performed in every hospital, which makes data sources very limited. In order to ensure the robust of the model, only subjects with complete clinical data were included, which made us must eliminate a lot of patients. With the increasement of clinical data, we look forward to optimizing our model with larger data sets soon. Second, the lack of an independent external validation set may prevent us from objectively evaluating our model. However, we randomly selected 30% of the data from the total population as the test set, which also ensures the independence of the validation data. Third, classification decision tree is only a kind of weak classifier in machine learning. But it belongs to a classic machine learning model, which is different from the "black box" model such as random forest and neural network, and its decision process can be clearly displayed, which is also important for clinicians. Finally, all of our data are from mainland China and only Chinese population is included in our study, which may result in our model failing to accurately predict other races such as Europeans, Americans and Africans. However, we believe that the tree model is a reliable method to reduce the deviation in estimating GFR. This method can also be applied to other populations, and the adjustment of tree model parameters in different populations may be a new problem in the future.

In summary, it is a novel approach to using a classification decision tree to select estimation equation and to estimate the GFR. We used machine learning methods combined with the advantages of multiple estimation equation to obtain a more accurate estimate of GFR. Taken together, this study provides an optimized way of machine learning that can efficiently select the appropriate equation and estimate GFR more accurately, which will help nephrologists precisely assess renal function in CKD patients.

## Methods

### Study design and subjects

This is a retrospective study. We searched the HIS system of the First Affiliated Hospital of Zhejiang Chinese Medicine University for all CKD patients who visited the hospital from December 1, 2018 to December 1, 2021 and underwent Gate’s method of ^99m^Tc-DTPA renal dynamic imaging to detect GFR. Subjects were included as follows: (1) clinically diagnosed with CKD; (2) ^99m^Tc-DTPA GFR was measured at the time of visit, and creatinine, cystatin C were available. Exclusion criteria included the following: (1) aged < 18 years; (2) underwent hemodialysis or peritoneal dialysis treatment within three months prior to the creatinine and cystatin C detection; (3) critical information, such as age and gender, was missing.

### Basic information collection

In this study, we collected the patients’ demographic data (age, sex, height, weight, systolic blood pressure, diastolic blood pressure, etc.), conditions related to renal disease (renal biopsy result, whether receiving glucocorticoid treatment and the use of immunosuppressants), previous history (cancer, diabetes, stroke, hyperuricemia, etc.), medication (diuretics, SGLT2i, RASi, etc.) and so on.

Du Bois equation is used to calculate the body surface area (BSA).

$$BSA={weight}^{0.425}*{height}^{0.725}*0.007184$$

### Laboratory and GFR measurements

Serum creatinine level was detected by sarcosine oxidase method with reagents purchasing from Zhongsheng Beikong Biotechnology Co., LTD. (92,644,093). Cystatin C was detected by latex enhanced immunoturbidimetry with reagents purchasing from Zhejiang Content Biotech Co., Ltd. (20,210,901). 24-hour Urinary Protein Quantity was detected by pyrogali-molybdic method with reagents purchasing from Beijing Leadman Biochemistry Co., Ltd. (21,011,107). The detection instrument is Abbott's ARCHITECT C16000 automatic biochemical analysis system (c16000659), and the specific detection is completed by the Clinical laboratory of Zhejiang Hospital of Chinese Medicine.

GFR was measured by Gate’s method of ^99m^Tc-DTPA renal dynamic imaging^34,35^, which used Single photon emission computed tomography scanner (INFINIA 17261, GE Healthcare). ^99m^Tc-DTPA was used as renal dynamic imaging agent with a dose of 185 MBq. ^99m^TC-DTPA renal dynamic imaging was performed in supine position and collected in posterior position. ^99m^Tc-DTPA 185 MBq was injected intravenously, and the collection procedure was started at the same time. Both kidneys were collected continuously. Low energy collimator, window width 20%, matrix 64 × 64, energy peak 140 keV, magnification 1–1.5. Dynamic collection was carried out for 31 min. Blood perfusion phase was collected for 1 min at 2 s/frame, and functional phase was collected for 30 min at 15 s/frame. The radioactivity count of the syringe was measured before and after injection. After the imaging was completed, the left and right kidneys and the background were manually delineated using ROI technology to generate time-radioactivity curves and calculate glomerular filtration rate of both kidneys.

### Statistical analysis

#### Statistical description and data set split

All continuous variables were presented as the mean (P_25_–P_75_) and categorical variables were described as N (n %). All the patients were randomly divided into two groups: 70% subjects (362) into the training set and 30% (156) into the test set.

#### Selection of eGFR estimation equation

Based on the current research, we selected some international GFR estimation equations and some GFR estimation equations developed for Chinese people. The unit of creatinine in *Xiangya* equation is μmol/L, and the units of other equation are mg/dL. 1 mg/dl creatinine = 88.4 μmol/L.

Cockcroft Gault:

$$eGFR=\frac{\left(140-Age\right)*height}{Cr*72}*0.85\;(if\;female)$$

The GFR was calculated in mL/min, which was converted to mL /min per 1.73 m^2^ based on the patient's body surface area.

MDRD:

$$eGFR=170*{Cr}^{-0.999}*{Age}^{-0.176}*{Bun}^{-0.170}*{Alb}^{0.318}*0.762\;(if\;female)$$

Abbreviated MDRD:

$$eGFR=175*{Cr}^{-1.154}*{Age}^{-0.203}*0.742\;(if\;female)$$

Chinese modification MDRD:

$$eGFR=170*{Cr}^{-0.999}*{Age}^{-0.176}*{Bun}^{-0.170}*{Alb}^{0.318}*1.202*0.762\;(if\;female)$$

Chinese modification abbreviated MDRD:

$$eGFR=170*{Cr}^{-0.999}*{Age}^{-0.176}*1.202*0.762 \;\left(if\;female\right)$$

CKD-EPI(Cr):

*In male*

$$eGFR=141*{\left(\frac{Cr}{0.9}\right)}^{-0.411}*{0.993}^{Age}\;(if\;Cr \le 0.9)$$

$$eGFR=141*{\left(\frac{Cr}{0.9}\right)}^{-1.209}*{0.993}^{Age}\;(if\;Cr >0.9)$$

*In female*

$$eGFR=144*{\left(\frac{Cr}{0.7}\right)}^{-0.329}*{0.993}^{Age}\;(if\;Cr \le 0.7)$$

$$eGFR=144*{\left(\frac{Cr}{0.7}\right)}^{-1.209}*{0.993}^{Age}\;(if\;Cr >0.7)$$

CKD-EPI(CysC):

*In male*

$$eGFR=133*{\left(\frac{CysC}{0.8}\right)}^{-0.499}*{0.996}^{Age}\;(if\;CysC \le 0.8)$$

$$eGFR=133*{\left(\frac{CysC}{0.8}\right)}^{-1.328}*{0.996}^{Age}(if CysC >0.8)$$

*In female*

$$eGFR=133*{\left(\frac{CysC}{0.8}\right)}^{-0.499}*{0.996}^{Age}*0.932\;(if\;CysC \le 0.8)$$

$$eGFR=133*{\left(\frac{CysC}{0.8}\right)}^{-1.328}*{0.996}^{Age}*0.932\;(if\;CysC >0.8)$$

CKD-EPI(Cr-CysC):

*In male*

$$eGFR=135*{\left(\frac{Cr}{0.9}\right)}^{-0.207}*{\left(\frac{CysC}{0.8}\right)}^{-0.375}*{0.995}^{Age}\;(if\;CysC \le 0.8 \& Cr\le 0.9)$$

$$eGFR=135*{\left(\frac{Cr}{0.9}\right)}^{-0.207}*{\left(\frac{CysC}{0.8}\right)}^{-0.711}*{0.995}^{Age}\;(if\;CysC >0.8 \& Cr\le 0.9)$$

$$eGFR=135*{\left(\frac{Cr}{0.9}\right)}^{-0.601}*{\left(\frac{CysC}{0.8}\right)}^{-0.375}*{0.995}^{Age}\;(if\;CysC \le 0.8 \& Cr>0.9)$$

$$eGFR=135*{\left(\frac{Cr}{0.9}\right)}^{-0.601}*{\left(\frac{CysC}{0.8}\right)}^{-0.711}*{0.995}^{Age}\;(if\;CysC >0.8 \& Cr>0.9)$$

*In female*

$$eGFR=130*{\left(\frac{Cr}{0.7}\right)}^{-0.248}*{\left(\frac{CysC}{0.8}\right)}^{-0.375}*{0.995}^{Age}\;(if\;CysC \le 0.8 \& Cr\le 0.7)$$

$$eGFR=130*{\left(\frac{Cr}{0.7}\right)}^{-0.248}*{\left(\frac{CysC}{0.8}\right)}^{-0.711}*{0.995}^{Age}\;(if\;CysC >0.8 \& Cr\le 0.7)$$

$$eGFR=130*{\left(\frac{Cr}{0.7}\right)}^{-0.601}*{\left(\frac{CysC}{0.8}\right)}^{-0.375}*{0.995}^{Age}\;(if\;CysC \le 0.8 \& Cr>0.7)$$

$$eGFR=130*{\left(\frac{Cr}{0.7}\right)}^{-0.601}*{\left(\frac{CysC}{0.8}\right)}^{-0.711}*{0.995}^{Age}\;(if\;CysC >0.8 \& Cr>0.7)$$

Asian modified CKD-EPI(Cr):

*In male*

$$eGFR=149*{\left(\frac{Cr}{0.9}\right)}^{-0.415}*{0.993}^{Age}\;(if\;Cr \le 0.9)$$

$$eGFR=149*{\left(\frac{Cr}{0.9}\right)}^{-1.210}*{0.993}^{Age}\;(if\;Cr >0.9)$$

*In female*

$$eGFR=151*{\left(\frac{Cr}{0.7}\right)}^{-0.328}*{0.993}^{Age}\;(if\;Cr \le 0.7)$$

$$eGFR=151*{\left(\frac{Cr}{0.7}\right)}^{-1.210}*{0.993}^{Age}\;(if\;Cr >0.7)$$

BIS-2:

$$eGFR=767*{CysC}^{-0.610}*{Cr}^{-0.400}*{Age}^{-0.570}*0.870\;(if\;female)$$

MacIsaac:

$$eGFR=\frac{86.700}{CysC}-4.200$$

Ruijin:

$$eGFR=234.960*{Cr}^{-0.926}*{Age}^{-0.280}*0.828\;(if\;female)$$

Xiangya:

$$eGFR=2374.780*{Cr}^{-0.54753}*{Age}^{-0.25011}*0.8526126;(if\;female)$$

#### Construction of classification decision tree model

We compared the eGFR obtained from the 4 most widely used and accurate equations in Chinese population (*BIS-2*, *CKD-EPI(CysC)*, *CKD-EPI(Cr-CysC)* and *Ruijin*) with GFR measured by ^99m^Tc-DTPA to obtain eGFR estimation equation with the smallest deviation for each patient as the best matched estimation equation. Then, we constructed a classification decision tree model of the optimal estimation formula according to the age, sex, body surface area, BMI, whether having the unilateral nephrectomy, history of hypertension, diabetes, cardiovascular disease, cerebral infarction, cerebral hemorrhage, cancer, hyperuricemia, gout, edema, whether taking calcium dobesilate, SGLT2i, RASi, beprostaglandin sodium, glucocorticoids, immunosuppressants, diuretics, smoking history, drinking history, pathological results of kidney biopsy, creatinine, cystatin, 24-hour urine volume, 24-hour urine protein of training set patients. In the construction process of classification decision tree model, CRT algorithm was selected as the basic algorithm for our model construction, where entropy was used to represent the information purity of each leaf and nodes were disassembled according to the highest information increment (The source code and annotations for the model building process are available in Supplement 2).

#### Accurate evaluation of GFR estimation based on classification decision tree and comparison with traditional equations

We predicted the appropriate equation based on the classification decision tree, and then used the forecasting equation to calculate eGFR. We verified the accuracy of the GFR estimation based on the classification decision tree model in the training set and test set data respectively, and compared it with other 13 traditional estimation equations. We used Root Mean Square Error (RMSE) and Mean Absolute Error (MAE) to evaluate the estimation effect of the model in the training set and the test set respectively, and take the estimation effect of test set as our result.

$$RMSE=\sqrt{\frac{1}{n}\sum {({y}_{predicted}-{y}_{actual})}^{2}}$$

$$MAE=\frac{1}{n}\sum \left|{y}_{predicted}-{y}_{actual}\right|$$

#### Programming languages and packages

We sued R, version 3.5.3 (R Foundation for Statistical Computing, Vienna, Austria. https://www.R-project.org/.) for data analysis. We used *rpart* (Terry Therneau and Beth Atkinson (2019). rpart: Recursive Partitioning and Regression Trees. R package version 4.1–15.) and *ggplot2* (H. Wickham. ggplot2: Elegant Graphics for Data Analysis. Springer-Verlag New York, 2016.) packages to construct classification decision tree model and plot Bland–Altman diagram.

### Ethical approval

This study was approved by the Ethics Committee of the First Affiliated Hospital of Zhejiang Chinese Medicine University (2022-KL-030-01). We also confirm that all research processes are carried out in accordance with relevant guidelines and regulations and under the supervision of the ethics committee and other regulators. Because our study was a retrospective analysis using data from the hospital information system, intervention of the subjects was not involved. We hid all patient information at the beginning of the study, fully protect the rights and interests of patients. So, the ethics committee has approved that we can exempt informed consent.

## Supplementary Information

Supplementary Information 1.

Supplementary Information 2.

## Data Availability

All data, models generated or used during the study appear in the submitted article. The datasets used and/or analyzed during the current study are available from the corresponding author on reasonable request.
