# Supplementary material for: Construct a classification decision tree model to select the optimal equation for estimating glomerular filtration rate and estimate it more accurately
Source: Sci Rep. 2022 Sep 1;12:14877. doi: 10.1038/s41598-022-19185-6 (PMC9436941; doi:10.1038/s41598-022-19185-6)
Supplement: Supplementary file 1 — Supplementary Information 1. [file 41598_2022_19185_MOESM1_ESM.pdf]

Supplement Table 1. GFR estimated by decision tree model and traditional equations based on BSA standardized GFR (Mean (P_{25}-P_{75}))

Equation[a]	Training Set (n=362)	Test Set (n=156)	Total Population (n=518)
Cockcroft Gault	72.0(37.9-100.8)	64.6(30.7-85.9)	69.8(35.2-95.7)
MDRD	67.2(28.7-99.7)	59.9(25.42-84.78)	65.0(27.4-94.0)
abbreviated MDRD	26.4(11.5-38.6)	23.4(9.9-33.69)	25.5(10.9-36.6)
Chinese Modification MDRD	80.8(34.6-119.9)	72.0 (30.6-101.9)	78.2(32.9-113.0)
Chinese Modification abbreviated MDRD	34.4(15.0 -50.4)	30.6(12.9-43.9)	33.2(14.2-47.7)
CKD-EPI(Cr)	27.1(11.3-39.4)	23.9(9.3-33.4)	26.1(10.7-36.9)
CKD-EPI(CysC)	24.1(13.8-29.8)	24.2(13.2-28.8)	24.1(13.6-29.7)
CKD-EPI(Cr-CysC)	24.0(11.9-31.7)	22.7(10.7-29.9)	23.6(11.5-30.8)
Asian modified CKD-EPI(Cr)	28.5(11.9-41.5)	25.2(9.8-35.3)	27.5(11.3-39.0)
BIS-2	28.9(18.2-36.2)	27.6(15.8-34.2)	28.5(17.6-35.8)
MacIsaac	31.2(19.7-38.9)	31.3(19.3-38.7)	31.3(19.6-38.9)
Ruijin	31.2(16.7-43.1)	28.2(14.6-38.2)	30.3(16.0-41.8)
Xiangya	41.4(30.0-52.8)	38.8(27.1-49.0)	40.7(29.1-51.2)
Decision Tree Classifier	26.8(15.3-35.4)	25.3(13.6-32.1)	26.4(14.6-34.5)
sGFR[b]	28.7 (15.5-38.6)	26.6 (15.1-34.6)	28.1 (15.3-37.2)

a. All GFR estimation equations were converted to a uniform unit, mL /min per 1.73 m^2

b. sGFR: GFR was measured by ^{99m}Tc-DTPA, and the GFR was converted to 1.73m^2 standard body surface area based on the patient's body surface area.

Supplement Table 2. RMSE and MAE of various estimation equations

	RMSE			MAE		
	Training Set (n=362)	Test Set (n=156)	Total Population (n=518)	Training Set (n=362)	Test Set (n=156)	Total Population (n=518)
Cockcroft Gault	56.0	50.4	54.4	44.2	38.7	42.5
MDRD	55.5	48.8	53.6	40.7	35.0	39.0
abbreviated MDRD	14.7	12.3	14.0	10.6	9.6	10.3
Chinese Modification MDRD	71.9	63.7	69.5	53.6	46.3	51.4
Chinese Modification abbreviated MDRD	19.2	15.9	18.2	13.5	11.7	12.9
CKD-EPI(Cr)	15.0	12.7	14.3	10.8	9.9	10.6
CKD-EPI(CysC)	15.1	14.0	14.8	10.5	9.5	10.2
CKD-EPI(Cr-CysC)	14.2	12.8	13.8	10.0	9.3	9.8
Asian modified CKD-EPI(Cr)	15.6	13.2	14.9	11.2	10.1	10.9
BIS-2	12.8	11.9	12.5	9.3	8.8	9.1
MacIsaac	15.3	15.0	15.2	11.0	10.8	10.9
Ruijin	13.9	11.6	13.2	9.9	8.9	9.6
Xiangya	17.7	16.2	17.3	14.9	13.9	14.6
Decision Tree Classifier	12.8	12.2	12.6	8.7	8.5	8.6

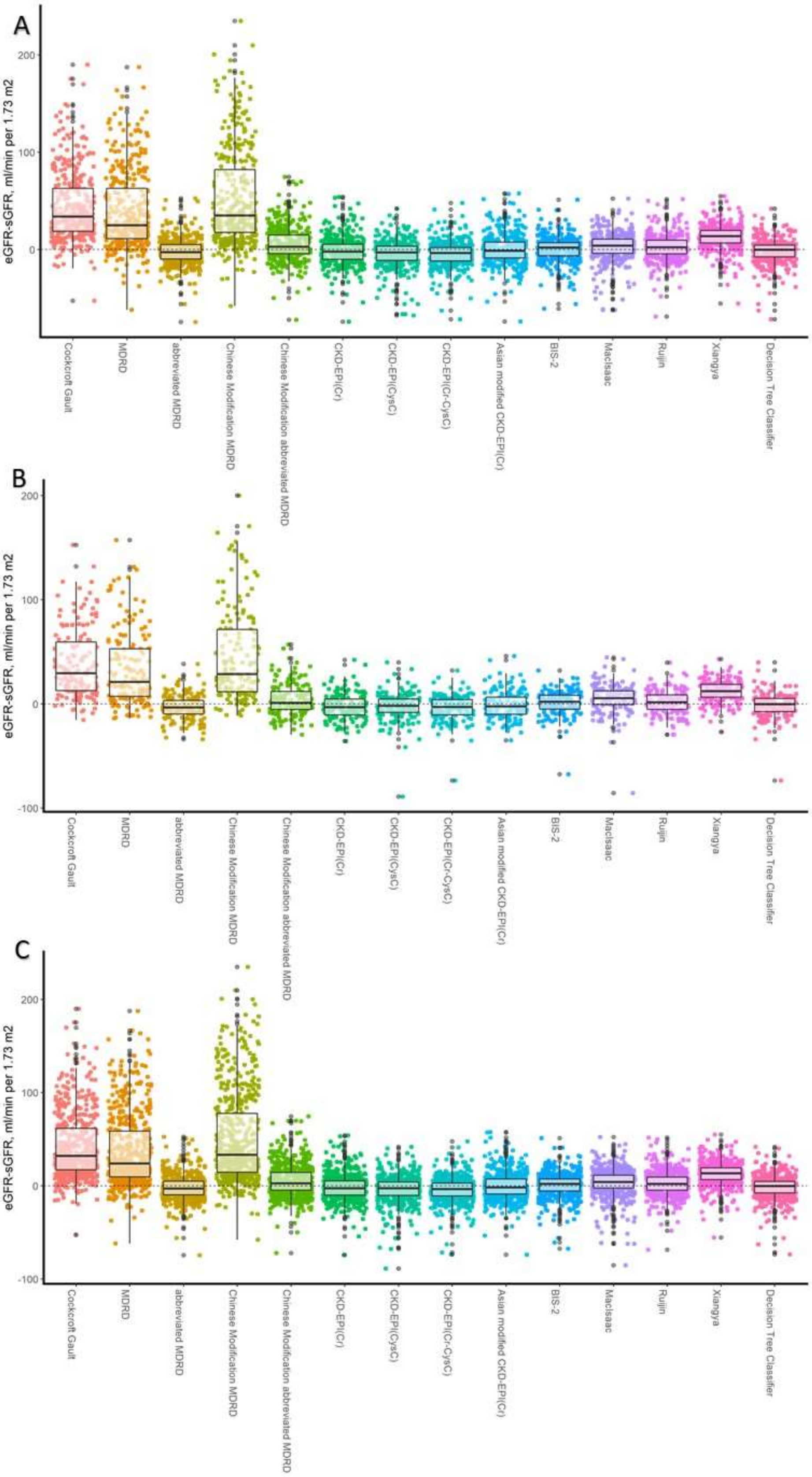

Supplement Figure 1. Variations in estimates of GFR in different equations| A: Training set; B: Test set; C: Total population; a: eGFR: Glomerular filtration rate was estimated based on an equation or model; b: sGFR: GFR was measured by ^{99m}Tc-DTPA, and the GFR was converted to 1.73m^2 standard body surface area based on the patient's body surface area.

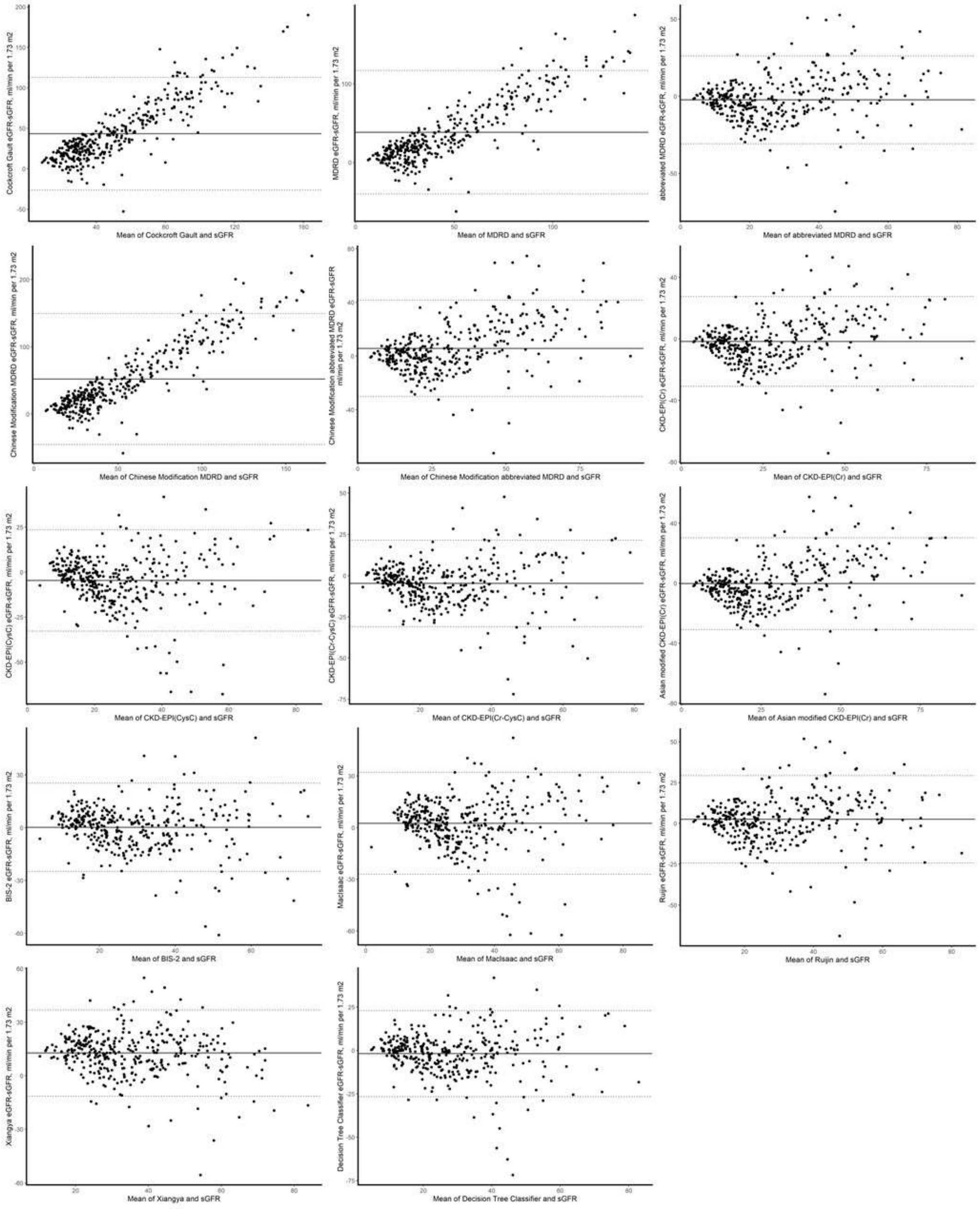

Supplementary Figure 2. Bland-Altman diagram for the training set

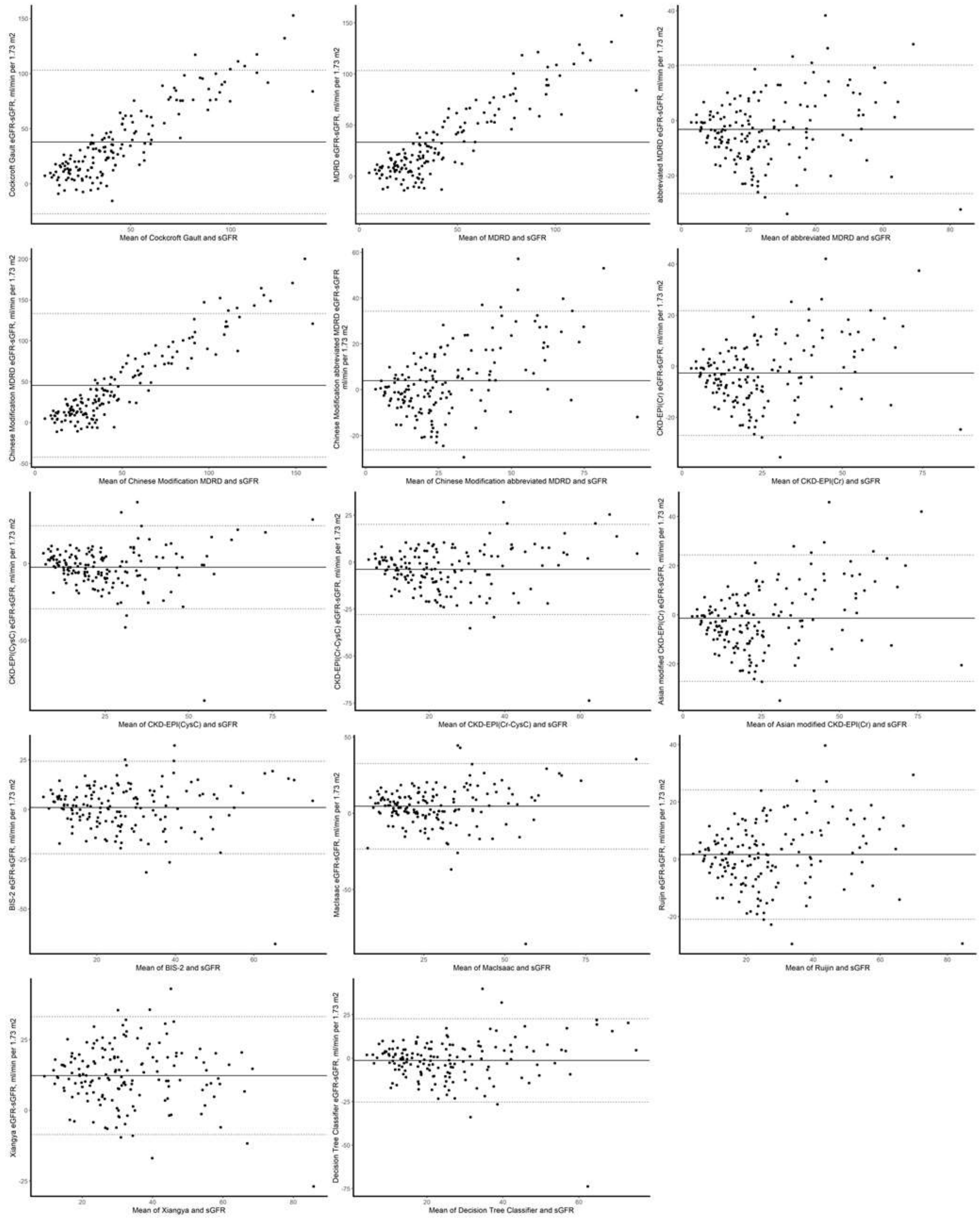

Supplementary Figure 3. Bland-Altman diagram for the test set

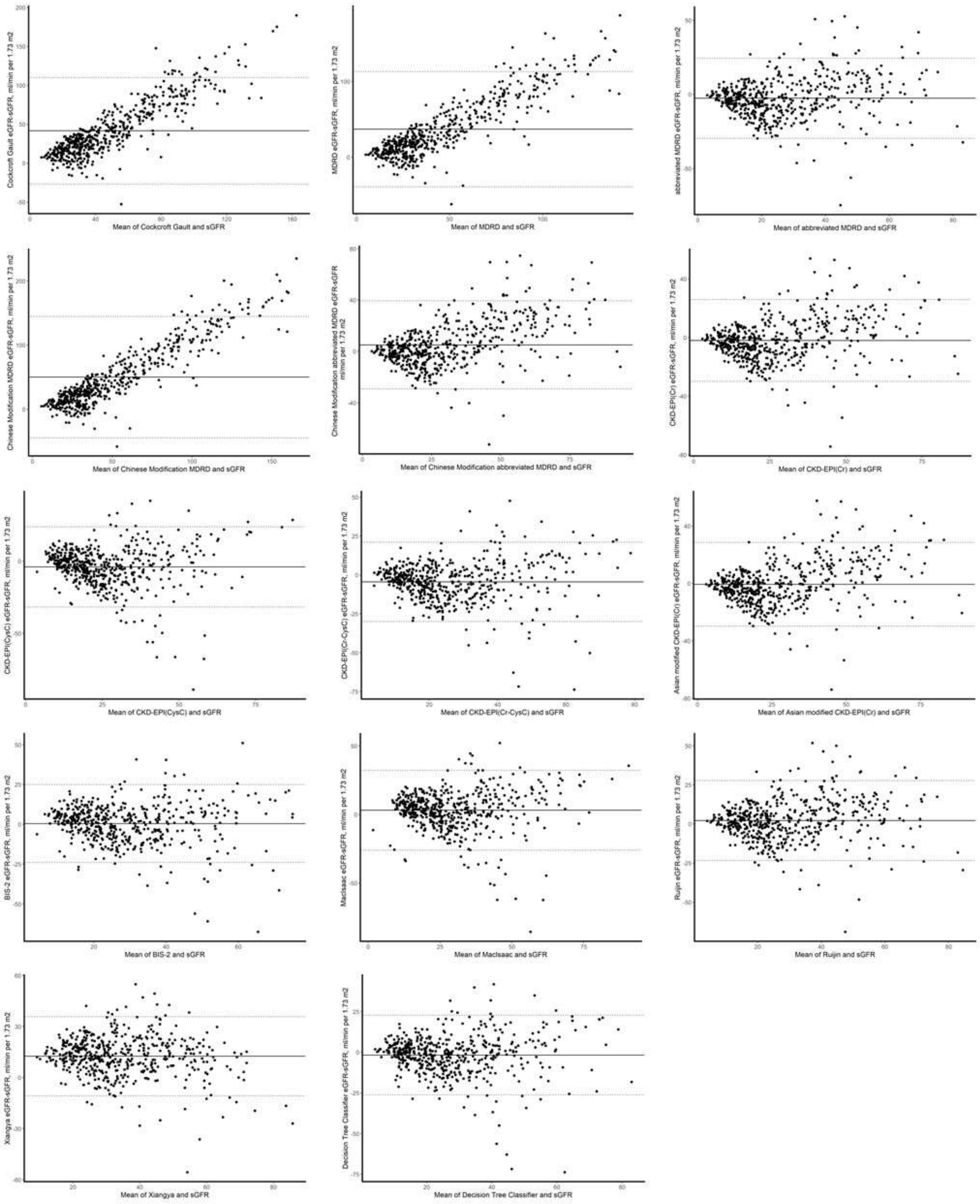

Supplementary Figure 4. Bland-Altman diagram for the total population
