# Supplementary material for: Construct a classification decision tree model to select the optimal equation for estimating glomerular filtration rate and estimate it more accurately
Source: Sci Rep. 2022 Sep 1;12:14877. doi: 10.1038/s41598-022-19185-6 (PMC9436941; doi:10.1038/s41598-022-19185-6)
Supplement: Supplementary file 2 — Supplementary Information 2. [file 41598_2022_19185_MOESM2_ESM.pdf]

```
# Build the formula
foru <- formula(min_foum~calciumdobesilate+age+sex+hypertension+diabetes+
                SGLT2i+hypertension+diabetes+cardiovasculardisease+cerebralinfarction+
                cerebralhemorrhage+cancer+hyperuricemia+gout+edema+smoke+drink+
                MCD+IgAN+MsPGN+MPGN+MN+FSGS+HyperuricemicNephropathy+
DN+
                HSP+LupusNephritis+HyperuricemicNephropathy+PolycysticKidney+
                UrineVolume+UrinaryProteinQuantity+RASi+
                beprostaglandinsodium+glucocorticoids+immunosuppressants+diuretics+
                surface+BMI+nu_of_kid # whether having the unilateral nephrectomy
                 )


############# constructional model ###############
fit <- rpart(formula = foru,
             data = training.data, #training set datd
             method = 'class',
             parms = list(split = "information"), # entropy was used to represent the
information purity of each leaf and nodes were disassembled according to the highest
information increment
             control = rpart.control(minsplit = 20, # the minimum number of observations
that must exist in a node in order for a split to be attempted.
                                     minbucket = round(minsplit/3), # the minimum
number of observations in any terminal <leaf> node. If only one of minbucket or minsplit is
specified
                                     cp = 0.005, # complexity parameter. Any split that
does not decrease the overall lack of fit by a factor of cp is not attempted.
                                     maxcompete = 4, # the number of competitor splits
retained in the output. It is useful to know not just which split was chosen, but which variable
came in second, third, etc.
                                     maxsurrogate = 5, # the number of surrogate splits
retained in the output. If this is set to zero the compute time will be reduced, since
approximately half of the computational time (other than setup) is used in the search for
surrogate splits.
                                     usesurrogate = 2, # how to use surrogates in the
splitting process. For value 2 ,if all surrogates are missing, then send the observation in the
majority direction. A value of 0 corresponds to the action of tree, and 2 to the recommendations
of Breiman et.al (1984).
                                     xval = 10, # number of cross-validations.
                                     surrogatestyle = 0, # controls the selection of a best
surrogate. If set to 0 (default) the program uses the total number of correct classification for a
potential surrogate variable
                                     maxdepth = 30# Set the maximum depth of any node
of the final tree, with the root node counted as depth 0. Values greater than 30 rpart will give
nonsense results on 32-bit machines.
                                     )
             )
fit
```

```
# Complexity related data
printcp(fit)
plotcp(fit)
# Post-Pruning
# Determines a nested sequence of subtrees of the supplied rpart object by recursively snipping off the least important splits, based on the complexity parameter (cp).
fit_pruned <- prune(fit, cp = fit$cptable[which.min(fit$cptable[,'xerror']),'CP'])
print(fit_pruned)
#Save model
# fit_pruned_save <- fit_pruned
# Variable Importance
fit_pruned$variable.importance
# Variable importance diagram
barplot(fit_pruned$variable.importance)
```
